# Auxin is a long-range signal that acts independently of ethylene signaling on leaf abscission in *Populus*

**DOI:** 10.3389/fpls.2015.00634

**Published:** 2015-08-12

**Authors:** Xu Jin, Jorma Zimmermann, Andrea Polle, Urs Fischer

**Affiliations:** ^1^Department of Forest Genetics and Plant Physiology, Umeå Plant Science Centre, Swedish University of Agricultural SciencesUmeå, Sweden; ^2^Forest Botany and Tree Physiology, Georg-August University of GöttingenGöttingen, Germany

**Keywords:** *Populus*, auxin, ethylene, abscission, cell separation, PIN proteins

## Abstract

Timing of leaf abscission is an important trait for biomass production and seasonal acclimation in deciduous trees. The signaling leading to organ separation, from the external cue (decreasing photoperiod) to ethylene-regulated hydrolysis of the middle lamellae in the abscission zone, is only poorly understood. Data from annual species indicate that the formation of an auxin gradient spanning the abscission zone regulates the timing of abscission. We established an experimental system in *Populus* to induce leaf shedding synchronously under controlled greenhouse conditions in order to test the function of auxin in leaf abscission. Here, we show that exogenous auxin delayed abscission of dark-induced leaves over short and long distances and that a new auxin response maximum preceded the formation of an abscission zone. Several auxin transporters were down-regulated during abscission and inhibition of polar auxin transport delayed leaf shedding. Ethylene signaling was not involved in the regulation of these auxin transporters and in the formation of an abscission zone, but was required for the expression of hydrolytic enzymes associated with cell separation. Since exogenous auxin delayed abscission in absence of ethylene signaling auxin likely acts independently of ethylene signaling on cell separation.

## Introduction

In contrast to animal cells, most plant cells are tightly glued together by the middle lamella, such that even subtle positional changes to neighboring cells are impeded. During the life cycle of plants, however, many developmental processes require cell separation, e.g. anther dehiscence, floral organ abscission, pod shatter, radicle emergence and leaf abscission (Roberts et al., 2002; Lewis et al., 2006). Although the enzymatic activities required for the hydrolysis of middle lamellae and cell walls in various separation phenomena are thought to be similar, the triggers and therefore the upstream signaling are obviously diverse (Taylor and Whitelaw, 2001; Roberts et al., 2002; Fischer and Polle, 2010). While stress-induced and developmentally regulated organ abscission is relatively well studied little attention has been paid to the understanding of how seasonal cues are integrated to trigger the separation of organs from the plant body. In temperate climates the most remarkable cell separation process is the autumnal shedding of leaves from trees in fall. Despite of the paramount importance of leaf shedding as an adaptation to freezing and for nutrient cycling in forest ecosystems (Aponte et al., 2013; Zanne et al., 2014) our understanding of autumnal leaf abscission is limited.

Important roles in timing of abscission have been assigned to the plant hormones ethylene and auxin. La Rue (1936) showed that removal of the leaf blade induces abscission; but when auxin is applied to the site of removal, abscission is inhibited. Addicott and Lynch (1955), Addicott et al. (1955) and Louie and Addicott (1970) applied auxin to decapitated stems of various annual species, which resulted in premature abscission of cotyledons or leaves. They found that not the absolute concentration of auxin but the ratio between distal and proximal auxin was relevant for the timing of abscission (Addicott et al., 1955; Louie and Addicott, 1970). Lower auxin concentrations on the distal than on the proximal side of the abscission zone favored abscission, whereas relatively higher auxin concentrations on the distal side delayed abscission (Louie and Addicott, 1970). From these experiments, it was concluded that an auxin gradient spans the abscission zone and regulates the induction of abscission (Addicott et al., 1955). Although testing of the auxin gradient model has been proven difficult in absence of highly resolved auxin concentration measurements or appropriate auxin response reporters the importance of auxin has further been strengthened by genetic evidence from the model plant *Arabidopsis*. Since petioles and pedicels *Arabidopsis* do not develop functional abscission zones (Patterson, 2001), research has focused on abscission of floral organs and dehiscence. Mutations in the auxin response factors *ARF1* and *ARF2* cause delayed petal abscission (Ellis et al., 2005). Similarly, expression of *iaa*L, which inactivates auxin, or *AXR3-1*, which is a stabilized Aux/IAA variant causing auxin resistance, under the control of an abscission zone specific promoter delays abscission (Basu et al., 2013).

Ethylene has been shown to play an antagonistic role to auxin in abscission of various organs. In the ethylene-insensitive *Arabidopsis* mutants *ein2* and *etr1-1* abscission is delayed (Bleecker and Patterson, 1997; Patterson and Bleecker, 2004), while application of ethylene hastens abscission in various organs and species. In line with a promotive role of ethylene in cell separation, ethylene levels often increase shortly before organ separation and ethylene is sufficient to induce the expression of a polygalacturonase required for cell separation in tomato petioles (Hong et al., 2000; Jiang et al., 2008). Interestingly, the same polygalacturonase is inhibited by the exogenous application of auxin, underlying the suggested antagonistic effects of auxin and ethylene in abscission (Hong et al., 2000). A broadly accepted model of hormonal interaction during organ separation suggests that a depletion of auxin levels in the abscission zone renders cells more sensitive to ethylene, which in turn induces secretion of middle lamellae hydrolyzing enzymes (Estornell et al., 2013).

Although the physiology and transcriptional changes related to autumnal leaf abscission in trees have been subject to intense studies (Chen et al., 1997; Keskitalo et al., 2005; Street et al., 2006; Giovannelli et al., 2007), mechanistic understanding of autumnal abscission signaling, especially the role of auxin, is limited. Here, we describe an experimental system to induce leaf abscission in *Populus* synchronously and report that auxin is a plausible long-range signal regulating abscission that acts independently of ethylene signaling.

## Materials and Methods

### Plant Material and Growth Conditions

*PttPIN1b::GUS, PttPIN5b::GUS, PttWAT1::GUS* constructs were transformed into hybrid aspen (*Populus tremula* L. X *P. tremuloides* Michx.; clone T89). The *in vitro* growth conditions were according to Love et al. (2009). Briefly, trees were grown in clear polypropylene containers (height, 14 cm; diameter, 10 cm) with OS140ODS140 gas-exchange spore filters (Combiness) and cultured on Murashige and Skoog medium (2.2 g⋅l^-1^, pH 5.6, Duchefa) with Phytagel P8169 solidifying agent (2.7 g⋅l^-1^; Sigma–Aldrich). Temperature was 22/18°C (light/dark), photoperiod was 16 h, and light intensity was 90 μmol⋅m^-2^⋅s^-1^. The 3 to 4-weeks-old *in vitro* grown transgenic trees (height, 10–12 cm) were transferred in 2.5 l pots with the commercial soil–sand–fertilizer mixture (Yrkes Plantjord Kronmull; Weibulls Horto, Hammenhög, Sweden) and grew in glasshouse under 18-h photoperiod at 20°C : 15°C (light : dark). Trees were fertilized with 150 ml of 1% Rika-S (N/P/K 7:1:5; Weibulls Horto) once a week (Vahala et al., 2013). The *GH3::GUS* lines are in the *Populus × canescens* backgroud, a hybrid between *P. alba* L. × *P. tremula* L. (Teichmann et al., 2008). These lines were used for the exogenous auxin and auxin transport inhibitor applications (**Figures 2** and **4**). All the other experiments were conducted in the T89 background.

### Dark Induction and Treatments with Auxin and Auxin Inhibitors

For dark-induction experiments, fully expanded leaves with a petiolar angle of 75 to 90° from 1.5 to 2 m tall trees were selected. Leaf blades were covered with aluminum bags under standard greenhouse conditions. Control samples were bagged in transparent plastic bags of the same weight. Each bag was labeled with a unique code referring to the genotype, tree replicate, leaf number, and treatment. Trees were gently shaken once per day, the dropped bags collected and the identifiers recorded.

Lanolin paste (Sigma–Aldrich) containing either 100 μM IAA, 2, 4-D, or morphactin (CF, Sigma–Aldrich) were placed with the help of a disposable 100 μl plastic tip at the junction between stem and petiole. For distal IAA application the lanolin paste containng 500 μM IAA was applied to the junction between the petiole and leaf blade.

For auxin induction experiment (Supplementary Figure S1), *in vitro* grown young shoots with 5–6 leaves of the respective GUS lines were treated with auxin [1 μM 1-naphthaleneacetic acid (1-NAA, Sigma–Aldrich) in ½ MS-medium (pH 5.8, Duchefa)] for 3 h in darkness and washed with ½ MS-medium and assayed for GUS.

### Cloning of Promoter Sequences and Plant Transformation

Nine hundred to thousand bp long promoter fragments of *PttPIN1b, PttPIN5b*, and *PttWAT1* were PCR amplified from *T89* using the primer combinations, PromPttPIN1b for/PromPttPIN1b rev, PromPttPIN5b for/PromPttPIN5b rev, or PromPttWAT1 for/PromPttWAT1 rev, respectively (Supplementary Table S3). The PCR products representing promoter regions of *PttPIN1b, PttPIN5b*, or *PttWAT1* were subcloned into pGemTeasy and moved directionally as *Pst*I-*Sal*I, *Sal*I-*Eco*RI, or *Hind*III-*Bam*HI fragments into the vector pCambia1391Z (http://www.cambia.org), respectively. The resulting plasmids were transformed into *Agrobacterium tumefaciens* strain GV3101 (pMP90, pSoup) and used for stable transformations of the hybrid aspen clone T89 according to Nilsson et al. (1992). For all constructs at least five lines were selected and tested for GUS expression. Three lines each were analyzed in detail and representative GUS expression patterns are shown in **Figure 5**.

### Gene Expression Analyses

Total RNA was extracted from 3-mm-long petioles near the junction to the stem using RNeasy Plant Mini Kit (Qiagen). The extracted total RNA was quantified with a ND-1000 NanoDrop spectrophotometer (NanoDrop Technologies, Wilmington, DE, USA). Two micrograms of total RNA was used as a template for reverse transcription with the QuantiTect Reverse Transcription Kit (Qiagen). Equal amounts of first-strand cDNAs were used as templates for real-time PCR amplification using the following primer combinations*: qPttPIN1bforw/qPttPIN1brev*; *qPttPIN5bforw/qPttPIN5brev*, and *qPttWAT1forw/qPttWAT1rev.* The T89 actin gene, *Actin1*, was amplified using primer combination *qPttActin1forw/ qPttActin1rev* (Supplementary Table S3). Quantitative real-time PCR was performed using LightCycler^®^ 480 SYBR Green I Master (Roche Diagnostics GmbH, Mannheim, Germany) with a LC4800 (Roche Diagnostics) qPCR machine. *PttPIN1b, PttPIN5b* and *PttWAT1* transcript levels were quantified in relation to *Actin1* levels. Microarrays (61 k Affymetrix Poplar array) employing four biological replicates were performed by MFTServices (Tuebingen, Germany). Data analysis has been performed with the help of the Robin software package (Lohse et al., 2010).

### Histochemical Staining for GUS Activity

GUS expression patterns were determined in 3-mm-thick longitudinal median sections of leaf axils. Samples were infiltrated employing vacuum for 30 min with GUS buffer (50 mM sodium phosphate buffer (pH 7.2), 5 mM K_3_Fe(CN)_6_, 5 mM K_4_Fe(CN)_6_, 0.1% Triton X-100) containing 2 mM 5-bromo-4-chloro-3-indolyl b-D-glucuronic acid (Duchefa Biochemie bv, The Netherlands) and then incubated in the dark at 37°C for 3–18 h. Pigments were removed in 70% (v/v) ethanol with gentle shaking. GUS expression patterns were examined with a bright-field microscope (Zeiss, Auxioplan) at low magnification (×2.5, ×10) or a scanner (Epson Perfection V600 Photo).

## Results

### An Auxin Response Maximum Precedes the Formation of an Abscission Zone

**FIGURE 1:**
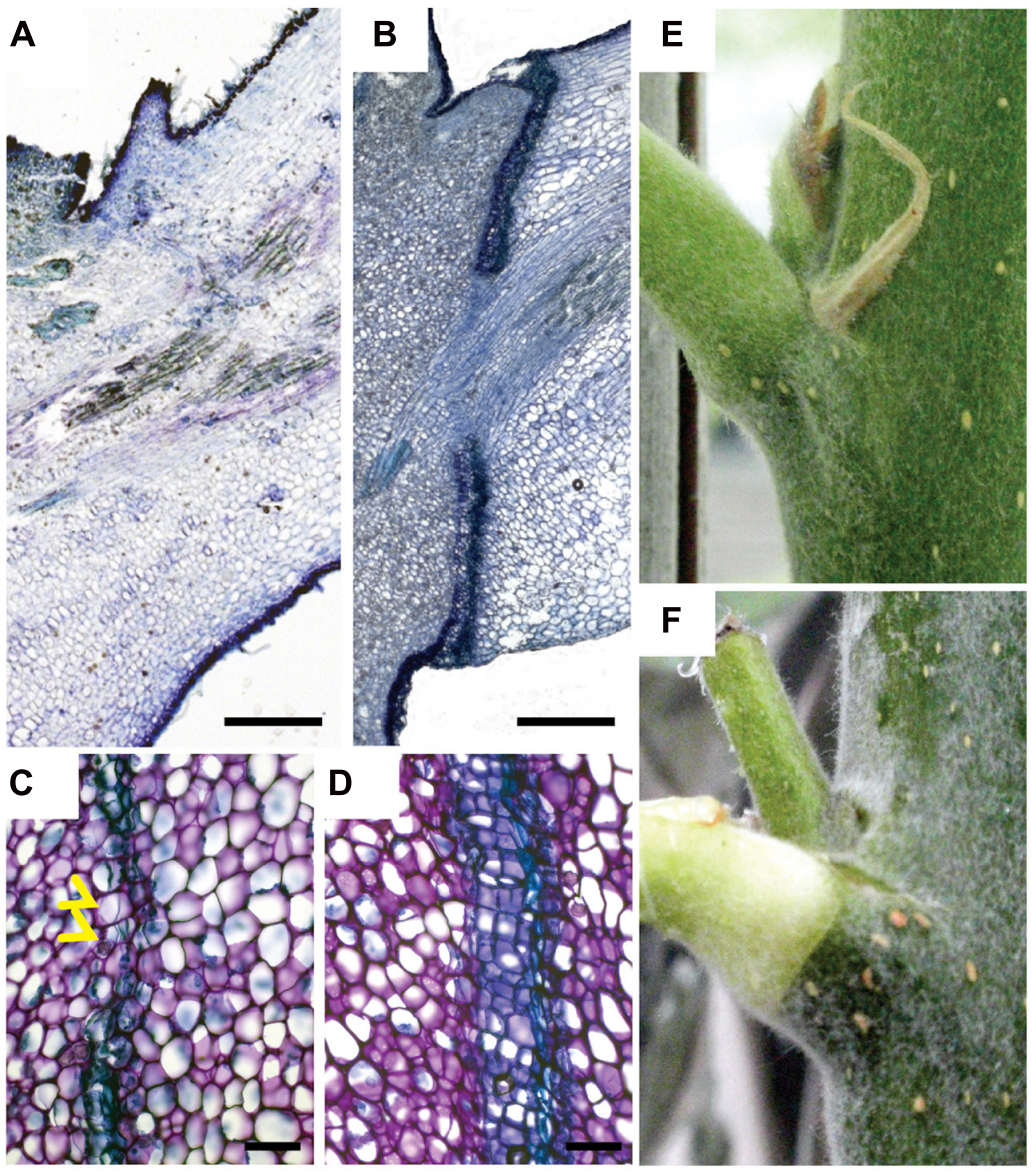
**A mobile signal from the leaf blade induces leaf abscission. (A–D)** Toluidine blue stained longitudinal median section of leaf axils. **(A)** No abscission zone visible in axil of non-shaded leaf blade. Blade was bagged in a transparent plastic bag for 9 days. **(B)** Mature abscission zone in an axil of a shaded leaf blade. Blade was covered for 9 days in aluminum foil. **(C,D)** Abscission zones 6 and 9 days after shading started, respectively. Yellow arrowheads point to dividing cells. **(E)** Leaf axil of a non-shaded blade. **(F)** Leaf axil of a blade shaded for 12 days. Chlorophyll distal to the abscission zone is degraded although leaf petiole was not shaded. Scale bars, 500 μm **(A,B)**; 100 μm **(C,D)**.

In *Populus*, gradual reduction of day length and temperature induces leaf abscission; whereas over-expression of phytochrome A is sufficient to prevent abscission under these conditions (Olsen et al., 1997). However, it remains unclear if shorter day length is sensed by individual leaf blades, apices or if it is rather a response of the whole plant to reduced photoperiod. Therefore, we tested if shading of the leaf blade is sufficient to induce abscission in greenhouse-grown *Populus* trees. To this end we covered blades but not petioles of fully expanded leaves with aluminum foil bags. Shading induced cell divisions in the leaf axil and the formation of typical abscission zones 6 and 9 days, respectively, after the treatment started. Although the petioles were not shaded strong de-greening of the petiole was observed (**Figure 1**). By contrast, leaves in transparent bags of the same tree did not develop an abscission zone, were not shed and their petioles did not de-greened during the same period of observation (**Figures 1A,E**). Taken together, these results suggest that reduced photoperiod is sensed in the leaf blade and a mobile signal transports this information from the blade to the abscission zone, where it induces the development of an abscission zone.

**FIGURE 2:**
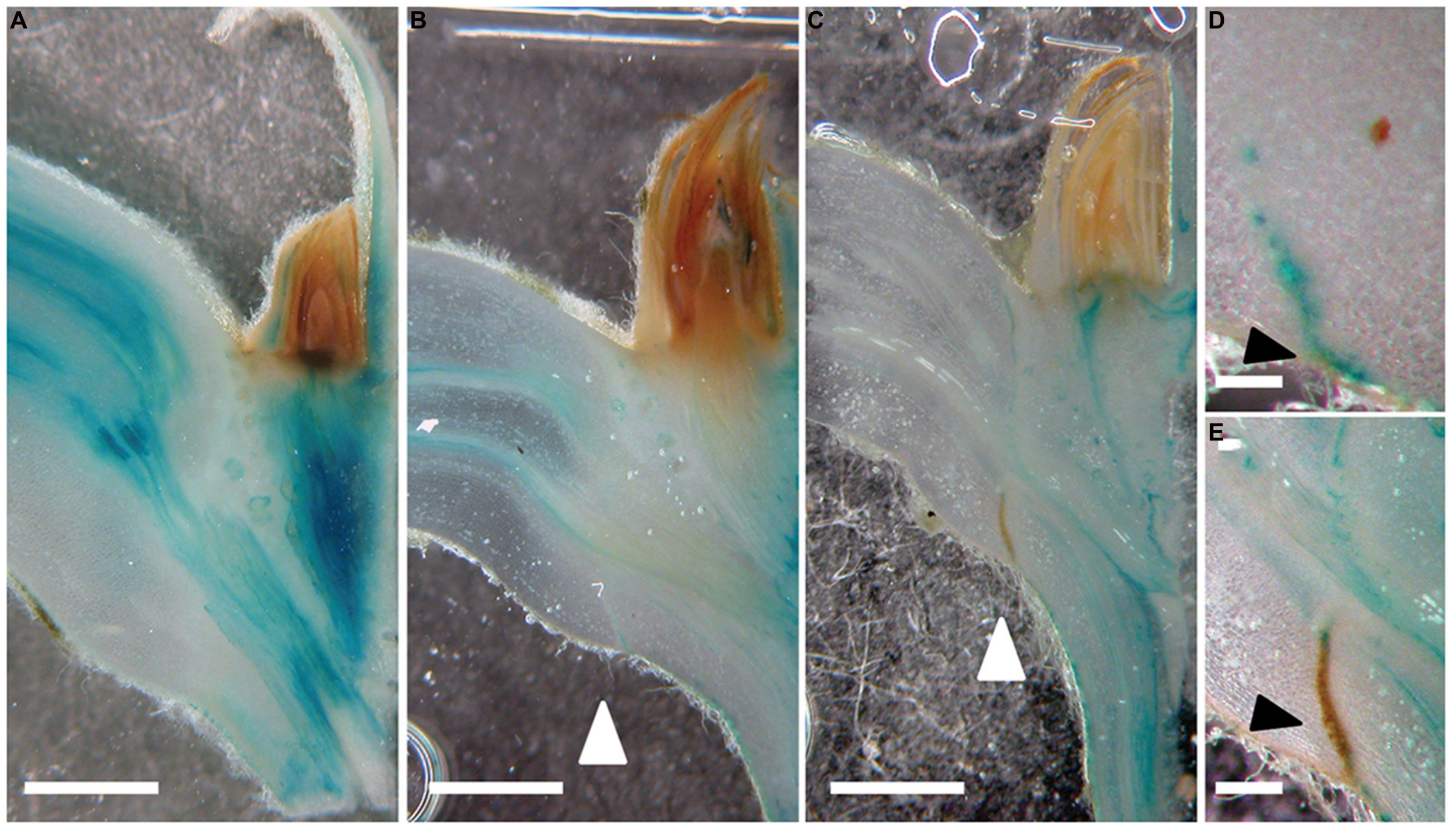
**A new auxin response maximum is established prior to the formation of an abscission zone. (A–E)** *GH3::GUS*; 0 **(A)**, 6 **(B,D)**, and 9 days **(C,E)** after shading started. White arrow heads point to the abscission zones. **(E)** Mature abscission zone appears in brown; GUS precipitate in blue. Scale bars correspond to approximately 1 mm **(A–C)**; 0,5 mm **(D,E)**. Black arrowheads point to the forming **(D)** and mature abscission zone **(E)**.

Previous work in explants of annual species indicated that auxin could contribute to a leaf blade-derived abscission inhibiting signal (Louie and Addicott, 1970). We therefore examined the expression patterns of the auxin response reporter *GH3::GUS* (Teichmann et al., 2008). In control petioles, *GH3::GUS* was strongest in the vascular tissues (**Figure 2A**). After 6 days of shading, this activity became weaker but at the lower (abaxial) side of the petiole a new auxin response maximum emerged, which gradually expanded to the upper (adaxial, after 9 days) side of the petiole preceding the formation and maturation of the abscission zone (**Figures 2B–E**).

**FIGURE 3:**
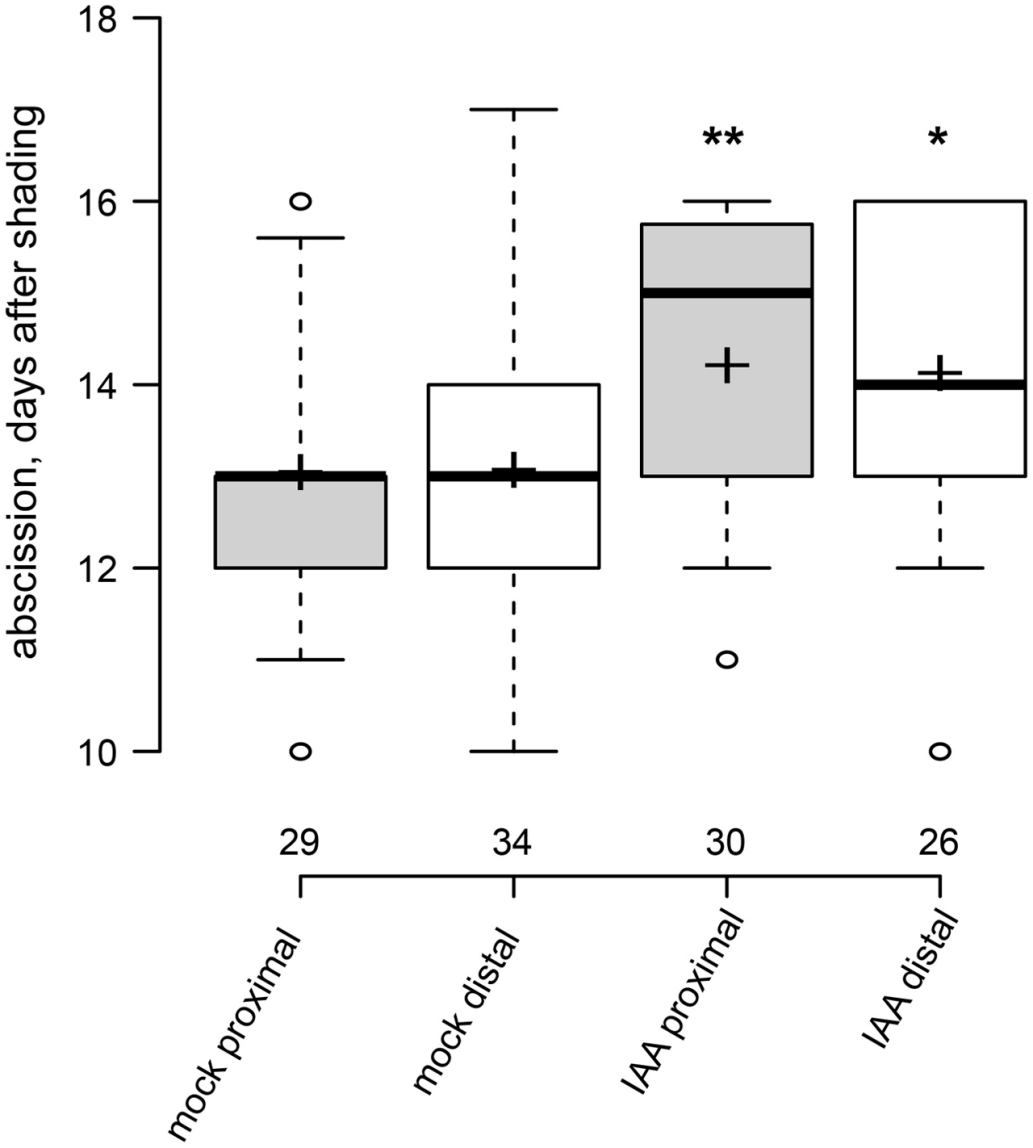
**Exogenous auxin can act over short and long distance to delay leaf abscission.** Box plot, center lines show the medians; box limits indicate the 25th and 75th percentiles; whiskers extend to 5th and 95th percentiles, outliers are represented by dots; crosses represent sample means. Number of observations indicated above the x-axis. ^∗^*p* < 0.05, ^∗∗^*p* < 0.01, *t*-test, control vs. treatment.

We then applied auxin (indole-3-acetic acid, IAA) in lanoline paste directly to the axils of intact leaves in order to test if auxin can delay abscission of shaded leaves (**Figure 3**). Auxin application to the very proximal end of the petiole delayed dark-induced leaf abscission highly significantly by approximately 1 day. In order to examine if auxin can also work as a long-distance signal, which is transported from the leaf blade to the axil, we applied auxin (IAA) to the most distal end of the petiole. Also in this case, abscission was significantly delayed supporting the idea that auxin not only acts locally but has the potential to be a long-distance signal in leaf abscission.

### Inhibition of Polar Auxin Transport Delays Formation of Abscission Zones

**FIGURE 4:**
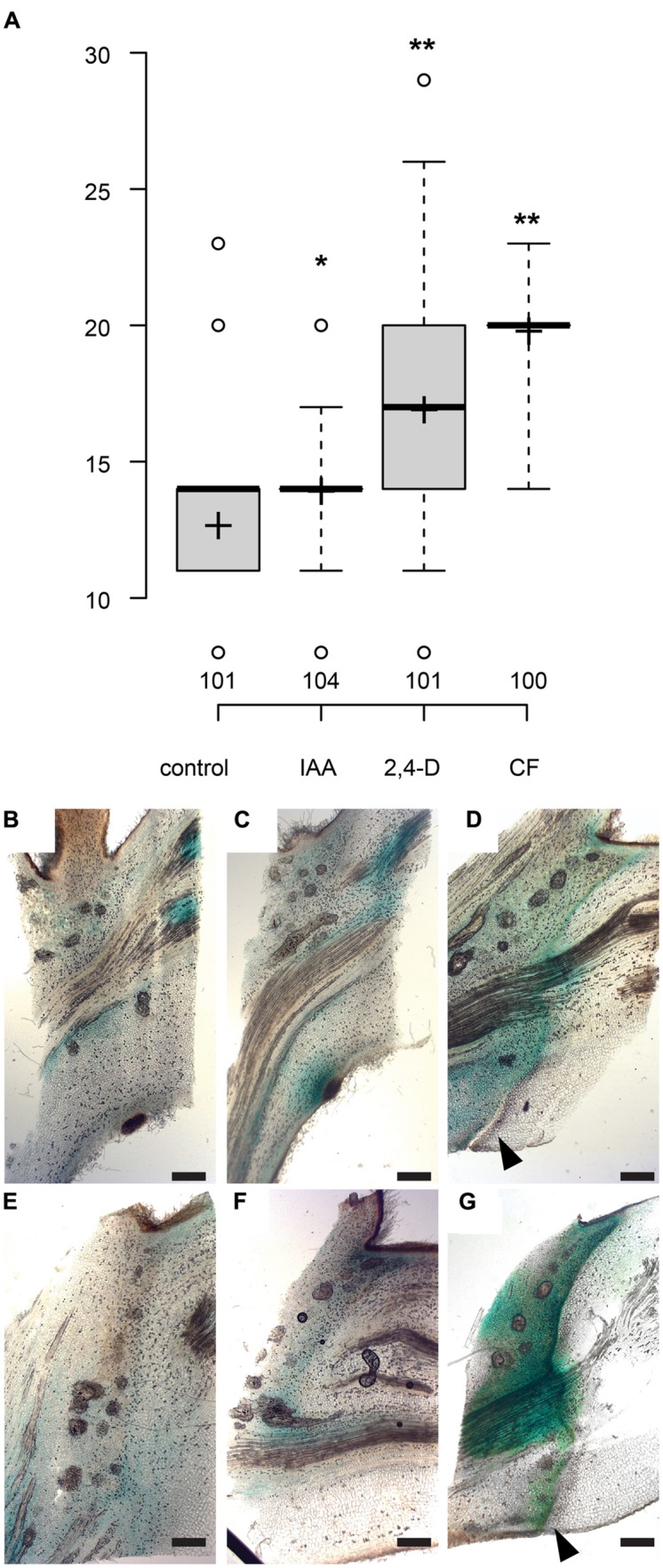
**The auxin transport inhibitor morphactin delays abscission. (A)** Box plot, center lines show the medians; box limits indicate the 25th and 75th percentiles; whiskers extend to 5th and 95th percentiles, outliers are represented by dots; crosses represent sample means. Number of observations indicated above the x-axis. ^∗^*p* < 0.05, ^∗∗^*p* < 0.01, *t*-test. **(B–G)** Formation of new auxin response maximum and abscission zone is delayed by morphactin. *GH3::GUS* (Teichmann et al., 2008). **(B–D)** Mock treatment, lanolin paste locally applied to abscission zone. **(E–G)** Lanolin paste containing 50 μM morphactin (CF) was applied to the leaf axils. 0 **(B,E)**, 6 **(C)**, 12 **(D,F**), and 18 days **(G)** after shading started; scale bars, 500 μm. **(D,G)** Arrowheads point to the abscission zone.

The polar auxin transport inhibitor morphactin (9-hydroxyfluorene-9-carboxylic acid, CF) has previously been show to delay leaf abscission in citrus (Goren et al., 1986). Local application of morphactin to the axil of shaded leaves delayed separation by approximately 7 days, to an even stronger extent as observed for the application of the auxin influx carrier substrate 2,4-D (**Figure 4A**). We then tested whether morphactin modulates auxin response in leaf axils. In mock-treated axils, 6 days after shading started, a new local *GH3::GUS* maximum appeared at the lower side of the petiole. After 9 days, in partly separated petioles, most of the auxin response reporter activity occurred on the proximal side of the abscission zone (**Figures 4B–D**). By contrast, in presence of morphactin formation of the new auxin response maxium as well as of the abscission zone were delayed and only became visible 18 days after shading started (**Figures 4E–G**). Hence, morphactin likely acts on the formation of the abscission zone by preventing the establishment of a new auxin response maximum.

### Auxin Transporters are Down-Regulated during Abscission

**FIGURE 5:**
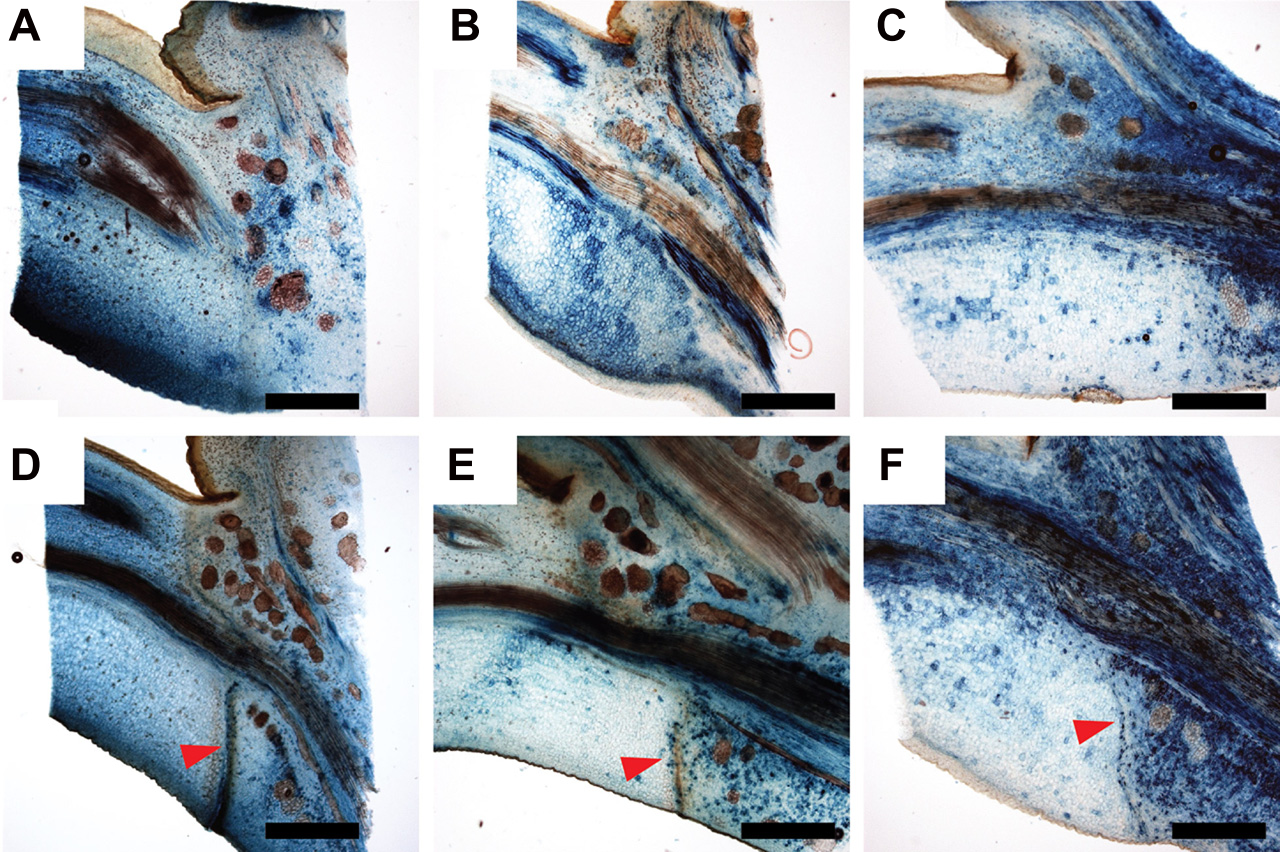
**Expression of *WAT1::GUS* and *PIN5b::GUS* is shifted to the proximal side of the abscission zone after shading of leaf blades. (A,D)** *PtrPIN1b::GUS*; **(B,E)** *PtrWAT1::GUS*; **(C,F)** *PtrPIN5b::GUS*. **(A–C)** axils of non-shaded leaf blades; **(D–F)** 9 days after shading started. Red arrowheads point to mature abscission zone. Scale bars, 1 mm.

In order to identify auxin transporters, which could be involved in the formation of a new auxin response maximum during abscission we performed a microarray experiment. Total RNA was isolated from dissected leaf axils 9 days after shading started and from axils covered with transparent plastic bags. Among the approximately 2400 differentially expressed genes auxin related transcripts were strongly overrepresented (Supplemental Dataset). Within the group of the 200 most strongly down-regulated genes several *AUX*/*IAA*s, *SAUR*s but also the putative auxin transporters *PtrPIN1b, PtrPIN5b*, and *PtrWAT1* were present (**Table 1**; data available at NCBI GEO, GSE69277). By contrast, none of the known putative auxin transporters were up-regulated. As its closest *Arabidopsis* homolog PIN1, PtrPIN1b localizes to the plasma membrane (Liu et al., 2014) and is most likely involved in intercellular auxin transport. On the other hand, the closest homologs of PtrPIN5b (PIN5) and PtrWAT1 (WAT1) localize in *Arabidopsis* to the endoplasmic reticulum and the tonoplast, respectively (Mravec et al., 2009; Ranocha et al., 2013), and are therefore expected to be involved in intracellular auxin transport and homeostasis. We isolated promoter fragments of *PttPIN1b, PttPIN5b, PttWAT1*, and used them to drive the GUS reporter gene. Expression of all reporter gene constructs was inducible by exogenous application of auxin (Supplementary Figure S1). In axils from non-shaded leaf blades, *pPttPIN1b::GUS* reporter activity was mainly associated with the vascular system across the entire axil (**Figure 5A**). Nine days after shading started additional *pPttPIN1b::GUS* activity was observed along the differentiating abscission zone (**Figure 5D**). Similarly, expression of the intracellular auxin transporters was most prominent in vascular tissues of axils from non-shaded leaf blades (**Figures 5B,C**). Shading led to strong, patchy expression of *PtrPIN5b::GUS* and *PtrWAT1::GUS* on the proximal side of the emerging abscission zone, whereas these genes were hardly expressed on the distal side (**Figures 5E,F**). These findings underline the importance of auxin transport in the formation of a new auxin maximum and the formation of an abscission zone.

**Table 1:** Genes involved in auxin transport and response are down-regulated in axils of shaded leaves.

Rank	ID	logFC	*P*-value	adj.*P*-Value	Closest At homolog	Process
8	PtpAffx.16882.2.S1_at	-5,8	6,7E-10	4,6E-06	*IAA19*	AUX/IAA
19	PtpAffx.25068.1.A1_at	-4,7	1,5E-07	7,2E-05	*IAA4*	AUX/IAA
20	PtpAffx.7696.4.S1_a_at	-4,7	1,8E-08	2,7E-05	*IAA4*	AUX/IAA
21	Ptp.128.1.S1_at	-4,7	2,7E-09	1,3E-05	*IAA14*	AUX/IAA
30	Ptp.6148.1.S1_at	-4,4	1,4E-06	1,9E-04	*PIN5*	Eﬄux
31	PtpAffx.7696.2.A1_a_at	-4,4	4,4E-07	1,1E-04	*IAA4*	AUX/IAA
36	PtpAffx.213779.1.S1_at	-4,3	9,7E-08	5,9E-05	*SAUR75*	Response
40	PtpAffx.21075.1.S1_at	-4,2	6,8E-07	1,3E-04	*SAUR14*	Response
45	PtpAffx.73583.1.S1_at	-4,0	8,7E-08	5,7E-05	*IAA4*	AUX/IAA
47	PtpAffx.102281.1.A1_at	-4,0	2,7E-06	2,7E-04	*SAUR75*	Respone
50	Ptp.127.1.S1_s_at	-4,0	7,6E-07	1,4E-04	*IAA4*	AUX/IAA
60	Ptp.1099.1.A1_at	-3,9	7,1E-07	1,4E-04	*GH3-10*	Homoestasis
74	PtpAffx.7696.4.S1_at	-3,6	1,7E-07	7,8E-05	*IAA4*	AUX/IAA
75	PtpAffx.204265.1.S1_at	-3,6	1,3E-04	3,0E-03	*SAUR14*	Response
84	Ptp.1274.1.S1_s_at	-3,5	8,2E-06	5,0E-04	*PIN1*	Eﬄux
94	PtpAffx.204337.1.S1_at	-3,3	1,5E-06	2,0E-04	*SAUR63*	Reponse
97	PtpAffx.123174.1.A1_at	-3,2	2,1E-06	2,3E-04	*IAA4*	AUX/IAA
103	PtpAffx.21896.2.S1_s_at	-3,2	3,7E-07	1,1E-04	*IAA13*	AUX/IAA
113	PtpAffx.249.95.S1_a_at	-3,0	1,2E-07	6,6E-05	*WAT1*	Homeostasis
161	Ptp.6738.1.S1_at	-2,7	2,2E-04	4,1E-03	*SAUR51*	Response
11	Ptp.6116.1.S1_at	4,5	1,1E-05	5,8E-04	*SUR2*	Homeostasis
13	PtpAffx.54125.1.A1_s_at	4,5	6,8E-06	4,5E-04	*SUR2*	Homeostasis
94	PtpAffx.210014.1.S1_at	3,1	1,8E-04	3,6E-03	*GH3-6*	Homeostasis
180	PtpAffx.221307.1.S1_at	2,6	4,0E-05	1,3E-03	*SAUR17*	Response

*RNA was extracted 9 days after dark-induction and gene expression was compared to the control treatment (leaves covered with transparent plastic bags of the same weight). Four biological replicates per treatment. Poplar 61 k Affymetrix array. The 200 most strongly up- or down-regulated were ranked and genes associated with auxin transport, response, or homeostasis were extracted from this list. Rank 1 corresponds to the most strongly up- and down-regulated genes, respectively.*

### Regulation of Auxin Transport during Abscission is Independent of Ethylene Signaling

**FIGURE 6:**
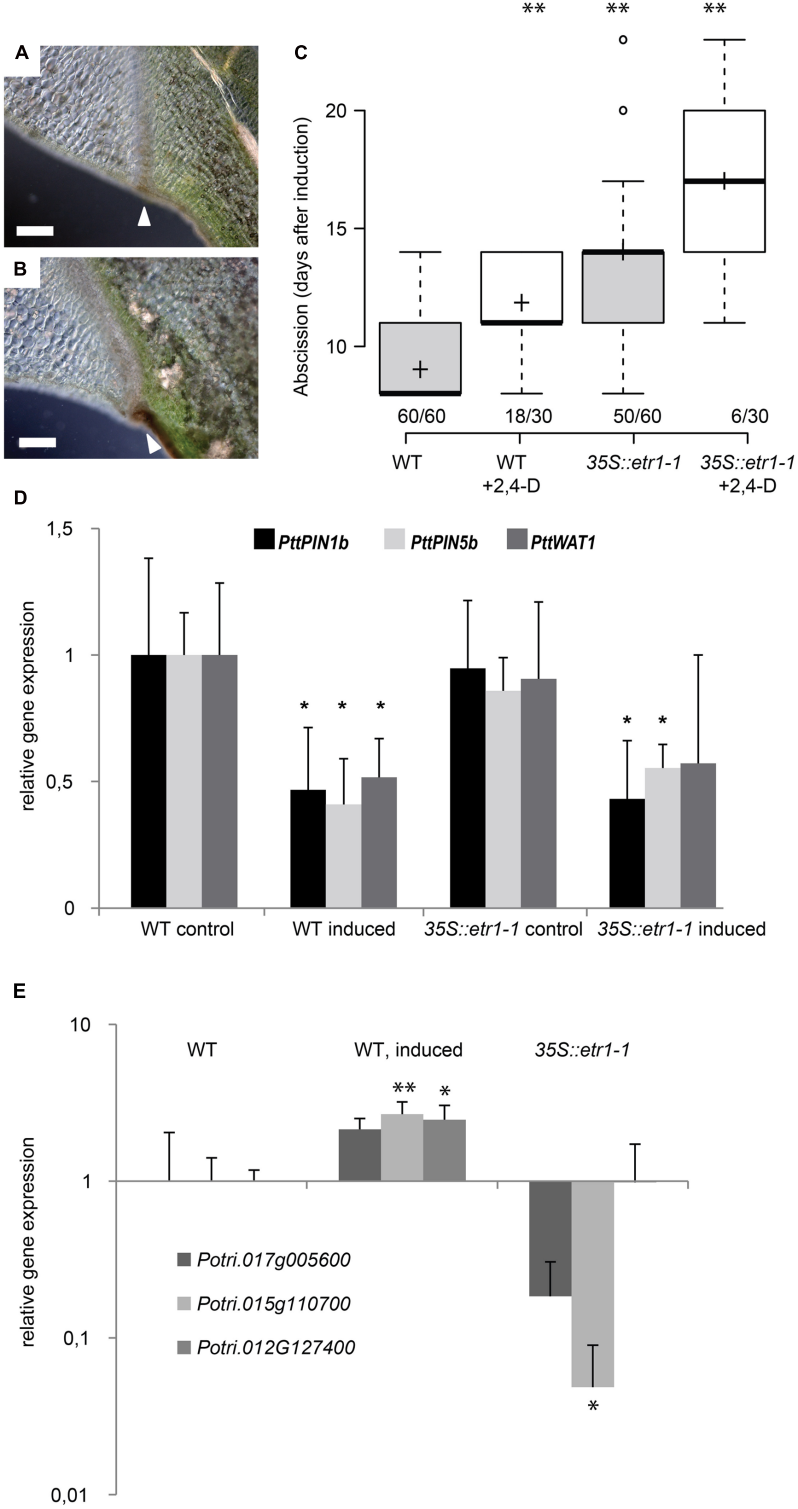
**Auxin acts independently of ethylene on leaf abscission. (A,B)** Abscission zone in wild-type **(A)** and *35S::etr1-1* **(B)** axils, 9 days after shading started. Scale bars, 200 μm. White arrowheads point to mature abscission zones. **(C)** Additive effects of 100 μM 2,4-D and inhibition of ethylene signaling (*35S::etr1-1*) on timing of leaf abscission. Box plot, center lines show the medians; box limits indicate the 25th and 75th percentiles; whiskers extend to 5th and 95th percentiles, outliers are represented by dots; crosses represent sample means. Number of observations indicated above the *x*-axis (number of shed leaves/total number of dark-induced leaves). ^∗∗^*p* < 0.01, *t*-test. **(D,E)** qRT-PCR, averages, and SD of three biological replicates. Normalized to *ACT1* expression. ^∗^*p* < 0.05, *t*-test, non-shaded versus shaded for 6 days. **(D)** Gene expression of auxin transporters. **(E)** Gene expression of pectin modifying enzymes. *Potri.017G005600* (homologs to the polygalacturonase encoding gene *ADPG1*), *Potri.015G110700* (homologs to the pectin methylesterase encoding gene *QRT1*) and *Potri.012G127400* (homologs to the pectin methylesterase inhibitor encoding gene *PME1*.

We then wondered if auxin acts through ethylene signaling on leaf abscission as it has been suggested previously (for review, Estornell et al., 2013). We first analyzed abscission in *35S::etr1-1* ethylene insensitive trees (Love et al., 2009). Expression of the dominant *Arabidopsis etr1-1* allele under the control of the *35S* promoter renders *Populus* trees insensitive to ethylene. *35S:: etr1-1* trees formed anatomically normally looking abscission zones after shading of leaf blades indicating that early steps of abscission could take place in absence of ethylene signaling (**Figures 6A,B**). *35S::etr1-1* leaves, however, separated later or not at all from the plant body (**Figure 6C**). We then tested if the expression of auxin transporter genes is reduced after shading in *35S::etr1-1* trees to a similar extent than in wild type. In wild-type leaf axils, *PttPIN1b, PttPIN5b*, and *PttWAT1* were significantly lower 6 days after shading started compared to non-shaded leaf axils (**Figure 6D**). Similar levels of dark-induced reduction in expression of *PttPIN1b* and *PttPIN5b* were observed in the ethylene-insensitive *35S::etr1-1* trees (**Figure 6D**). By contrast, close homologs of the pectin methylesterase *QUARTET1* and the polygalacturonase *ADPG1*, which are required for cell separation during microgenesis and dehiscence (Francis et al., 2006; Ogawa et al., 2009), were among the most strongly up-regulated genes after leaf shading and their up-regulation was dependent on ethylene signaling (**Figure 6E**; Supplementary Table S2). Together these findings suggest that the regulation of auxin transporters and the formation of an abscission zone are independent of ethylene signaling. In order to test if auxin acts on leaf abscission solely upstream of ethylene or in an independent parallel pathway we tested if auxin has an effect on the timing of abscission in *35S::etr1-1* axils of shaded leaves (**Figure 6C**). Exogenous auxin further delayed abscission and the number of leaves, which did not abscise, in an additive manner, as compared to mock treated *35S::etr1-1* axils, suggesting that auxin acts in an independent pathway, parallel to ethylene signaling, on leaf abscission.

## Discussion

Based on earlier work, an auxin gradient spanning the abscission zone has been proposed to regulate timing of organ separation (Addicott et al., 1955). Instead of an auxin response gradient we observed a discrete auxin response maximum, which precedes the formation of an abscission zone, indicating that auxin provides positional cues for the specification and/or differentiation of an abscission zone. Similar spatially discrete auxin maxima or gradients have been proposed to regulate the positioning of leaf and lateral root primordia as well as the zonation patterning in the cambium (Dubrovsky et al., 2008; Kierzkowski et al., 2013; Bhalerao and Fischer, 2014). In the cambium high auxin concentrations correlate with meristematic activity and cell division of protoplasts requires the presence of auxin (Bhalerao and Fischer, 2014). Intriguingly, formation of the abscission zone not only involves specialization of cells but also in many cases cell proliferation. In contrast to an auxin response maximum, which precedes the formation of an abscission zone, Sorefan et al. (2009) reported that a local auxin response minimum is required for differentiation of the separation layer of the valve margin. At the site of reduced auxin response secondary walls are deposited, which are instrumental during the separation process. Interestingly, a strong auxin response is detected in the replum, the tissue between valve margins, coincidentally, overlapping with the region where cell divisions occur during the formation of an abscission zone (Wu et al., 2006; Sorefan et al., 2009). Thus, the mode of auxin action might be similar in abscission zones as suggested for the cambium, high concentrations permit cell division, below a certain threshold cell expansion and differentiation are favored.

Recently, an auxin response maximum has been observed in the abscission zone of *Arabidopsis* petals (Basu et al., 2013). In auxin influx carrier mutants, petal break strength is increased and therefore *AUX1* and its paralogs might be involved in the formation of an auxin concentration maximum in the abscission zone (Basu et al., 2013). In mutants of the auxin eﬄux carrier PIN4, additional root cap layers are observed (Friml et al., 2002). In wild-type, the outermost tier of the root cap is continuously shed from the root. The *pin4* phenotype is either a consequence of over-proliferation of root cap cells or, more likely, of impaired cell separation. Our findings of delayed auxin maximum formation and abscission upon inhibition of polar auxin transport and the strong reduction of auxin carriers in dark-induced leaf axils are in support with the idea that auxin transport is instrumental for the establishment of an instructive auxin response maximum during abscission.

Interestingly, expression of *iaa*L, which conjugates auxin, under the control of an abscission zone specific polygalacturonase promoter results in reduced auxin response and increased petal break strength (Basu et al., 2013). Polygalacturonases are integral parts of cell wall remodeling during the separation process (Roongsattham et al., 2012) and it is likely that the promoter used to drive *iaa*L is specifically active during later phases of abscission rather than during the formation of an abscission zone. Consequently, relatively low auxin concentrations during the separation phase are sufficient to hasten abscission. Hence, auxin is likely to have a dual function during abscission in first providing positional information for the formation of the abscission zone and secondly as a signal, which regulates temporal aspects of the separation. Such a mode-of-action ensures that under high auxin concentrations an abscission zone can differentiate but separation cannot be initiated. As a consequence premature abscission, which can be deleterious for plant performance, might be prevented.

Ethylene induced reduction of auxin transport capacity in the midrib of poplar leaves correlates with leaf abscission (Riov and Goren, 1979). Furthermore, it has been suggested that reduced uptake of auxin into the cells of the abscission zone increases their sensitivity toward ethylene (Taylor and Whitelaw, 2001). We observed that formation of an abscission zone and the regulation of auxin transport in petioles of dark-induced leaves are independent of ethylene signaling. Importantly, auxin could further delay abscission of ethylene insensitive petioles. Therefore, auxin may act in parallel and independently of ethylene on the hydrolysis of middle lamellae. The existence of such an ethylene-independent, additional signaling pathway has been suggested earlier, based on the observation that petal abscission in the ethylene insensitive mutants *ein2* and *etr1-1* is only incompletely inhibited (Bleecker and Patterson, 1997).

Evidence for a non-cell-automonous signal, which provides positional information for the formation of an abscission zone, was first provided by the analyses of the MADS box transcription factor JOINTLESS in tomato. *jointless* mutants do not form abscission zones in pedicels (Mao et al., 2000). Interestingly, *JOINTLESS* function in in the clonal layer L3 (vasculature) is sufficient to rescue the *jointless* phenotype. This finding strongly suggests that JOINTLESS can act through a mobile factor on cells in the cortex and epidermis of the pedicel to induce the formation of an abscission zone (Szymkowiak and Irish, 1999). The establishment of a new auxin maximum prior to the maturation of the abscission zone and regulation of auxin transporters in the abscission zone after dark induction are arguments in favor of auxin to provide cues to position the abscission zone, similar as for the formation of primordia in apical meristems and during differentiation of cambial derivatives. Intriguingly, exogenous auxin is sufficient to induce ectopic expression of the peptide hormone INFLORESCENCE DEFICIENT IN ABSCISSION (IDA), which controls cell separation during floral organ abscission and lateral root primordia emergence (Aalen et al., 2013; Kumpf et al., 2013). Besides the functional analyses of the identified auxin transporters *PtrPIN1b, PtrPIN5b* and *PtrWAT1*, future directions of research should include the determination of subcellular localization of auxin eﬄux carriers in the petiole. Subsequent modeling of auxin distribution could reveal if a scenario of auxin providing positional information to the cells in the petiole is plausible.

## Author contributions

XJ, JZ, and UF performed and analyzed the experiments. AP and UF designed the research and edited the manuscript. XJ and UF wrote the manuscript.

## Conflict of Interest Statement

The authors declare that the research was conducted in the absence of any commercial or financial relationships that could be construed as a potential conflict of interest.
