# Supplementary material for: Auxin is a long-range signal that acts independently of ethylene signaling on leaf abscission in Populus
Source: Front Plant Sci. 2015 Aug 12;6:634. doi: 10.3389/fpls.2015.00634 (PMC4532917; doi:10.3389/fpls.2015.00634)
Supplement: Supplementary file 1 [file Presentation_1.PDF]

Supplementary Material

Auxin is a long-range signal that acts independently of ethylene signaling on leaf abscission in Populus

Xu Jin[1,2], Jorma Zimmermann[1,3], Andrea Polle[2] and Urs Fischer[1,2,*]

[1] Department of Forest Genetics and Plant Physiology, Umeå Plant Science Centre, Swedish University of Agricultural Sciences, Umeå, Sweden
[2] Forest Botany and Tree Physiology, Georg-August University of Göttingen, Göttingen, Germany,

*** Correspondence:** Urs Fischer, Department of Forest Genetics and Plant Physiology, Umeå Plant Science Centre, Swedish University of Agricultural Sciences, Umeå, Sweden.

urs.fischer@slu.se email@uni.edu

[3]Present address: Plant Ecology and Ecosystem Research, Georg-August University Göttingen, DE-37073, Göttingen, Germany

Supplemental Material

Supplemental Figure 1. Expression of auxin transporters is inducible by exogenous NAA.
Supplemental Table1. Closest Arabidopsis homologs of *Populus* auxin transporters.
Supplemental Table2. Gene expression of pectin modifying enzymes.
Supplemental Table3. Primer sequences.
Supplemental Dataset. 61 k Affymetrix Poplar array, axils from shaded versus non-shaded leaves.

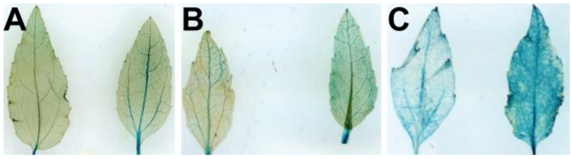

Supplemental Figure 1. Expression of auxin transporters is inducible by exogenous NAA. Histochemical staining for GUS activity in transgenic Populus plants expressing the *GUS* reporter gene under the control of putative promoters (1000-bp 5′ of ATG) of *PtrPIN1b*, *PtrPIN5b* and *PtrWAT1*. Samples were stained after 3 h incubation in ½ MS liquid medium without and with 1µM NAA. Images are representative of at least three independent transgenic lines. For each line, at least five leaves were recorded. (A) *PtrPIN1b::GUS*, (B) *PtrPIN5b::GUS*, (C) *PtrWAT1b::GUS*. (A-C) control, left side; NAA treatment, right side. Leaf blades are approximately 1 cm long.

Supplemental Table 1. Closest Arabidopsis homologs of Populus auxin transporters.

Gene name	Gene model (V3)	Arabidopsis gene locus	Amino Acids	Protein Size in kD
PtrPIN1b	*Potri.015G038700*	*At1g73590*	614	67.4
PtrPIN5b	*Potri.013G087000*	*At5g16530*	346	37.9
PtrWAT1	*Potri.005G233600*	*At1g75500*	384	41.9

Supplemental Table 2. Gene expression of pectin modifying enzymes

Rank	ID	logFC	P.Value	adj.P.Val	closest At homolog	alias
22	PtpAffx.30927.1.A1_at	4,1	1,2E-05	6,1E-04	AT5G51520	Potri.012G127400
41	PtpAffx.225327.1.S1_at	3,6	8,0E-07	1,4E-04	AT3G59850	Potri.017G005600
48	Ptp.4202.1.S1_at	3,5	6,3E-06	4,3E-04	AT1G69940	Potri.015G110700

Supplemental Table 2. Gene expression of pectin modifying enzymes. RNA was extracted 9 days after dark-induction and gene expression was compared to the control treatment (leaves covered with transparent plastic bags of the same weight). 4 biological replicates per treatment. Poplar 61 k Affymetrix array. The 200 most strongly up- or down-regulated were ranked. Rank 1 corresponds to the most strongly up-regulated gene. Gene expression of the three most strongly up-regulated of pectin modifying enzymes is shown. *Potri.017G005600* (homologues to the polygalacturonase encoding gene *ADPG1*), *Potri.015G110700* (homologues to the pectin methylesterase encoding gene *QRT1*) and *Potri.012G127400* (homologues to the pectin methylesterase inhibitor encoding gene *PME1*.

Supplemental Table 3. Primer sequences

Designation	Sequence
PromPtrPIN1b for	5'-GCCTGCAGGAAATAAATAGAATAAGAAC-3'
PromPtrPIN1b rev	5'-GCGTCGACCTTTGGTTACTTTTTGGATC-3'
PromPtrPIN5b for	5'-GCGTCGACTCCTTATTCGAAGATTAAAA-3'
PromPtrPIN5b rev	5'-GCGAATTCCTTTCTTTCTTTTCTTTTTC-3'
PromPtrWAT1 for	5'-GCAAGCTTGGGGTTAAAGTATACTATTG-3'
PromPtrWAT1 rev	5'-GCAAGCTTGGGGTTAAAGTATACTATTG-3'
GUS SEQ forw.	5'-GACGACTCGTCCGTCCTGTA-3'
GUS SEQ rev.	5'-CCCACACTTTGCCGTAATGAGT-3'
PtrPIN1 forw. qPCR	5'-GGACAGAGAGAAAACCAGGAGGA-3'
PtrPIN1 rev. qPCR	5'-TGTTGGAGGCATTGGTTTTGG-3'
PtrPIN5 forw. qPCR	5'-CAGGACTTGGTTTTTCATCCAACA-3'
PtrPIN5 rev. qPCR	5'-AAAAGCCCAAACAAGGCCAAT-3'
PtrWAT1 forw. qPCR	5'-GGCATTGGAAAAGGCTGCAA-3'
PtrWAT1 rev. qPCR	5'-ACGAAGGGAGCAGTGGCTGA-3'
017G005600_LI qPCR	5'-CCGGAAACAGCCCTAACACG-3'
017G005600_RI qPCR	5'-GATTCCATGGCCAGGTCCAC-3'
015G110700_LII qPCR	5'-CCCTGGTGGATGGTCCGATA-3'
015G110700_RII qPCR	5'-GCAGCCATTTGGAACCTTCG-3'
012G127400_LI qPCR	5'-ATGTGCAGCTTTCCTTGGCA-3'
012G127400_RI qPCR	5'-GGTCACAGTTAACGCTGTTCTGC-3'
PtrActin1 forw.	5'-CGATGCCGAGGATATTCAAC-3'
PtrActin1 rev.	5'-ACCAGTGTGTCTTGGTCTACCC-3'
