# Supplementary figures and images for: Auxin is a long-range signal that acts independently of ethylene signaling on leaf abscission in Populus
Source: Front Plant Sci. 2015 Aug 12;6:634. doi: 10.3389/fpls.2015.00634 (PMC4532917; doi:10.3389/fpls.2015.00634)

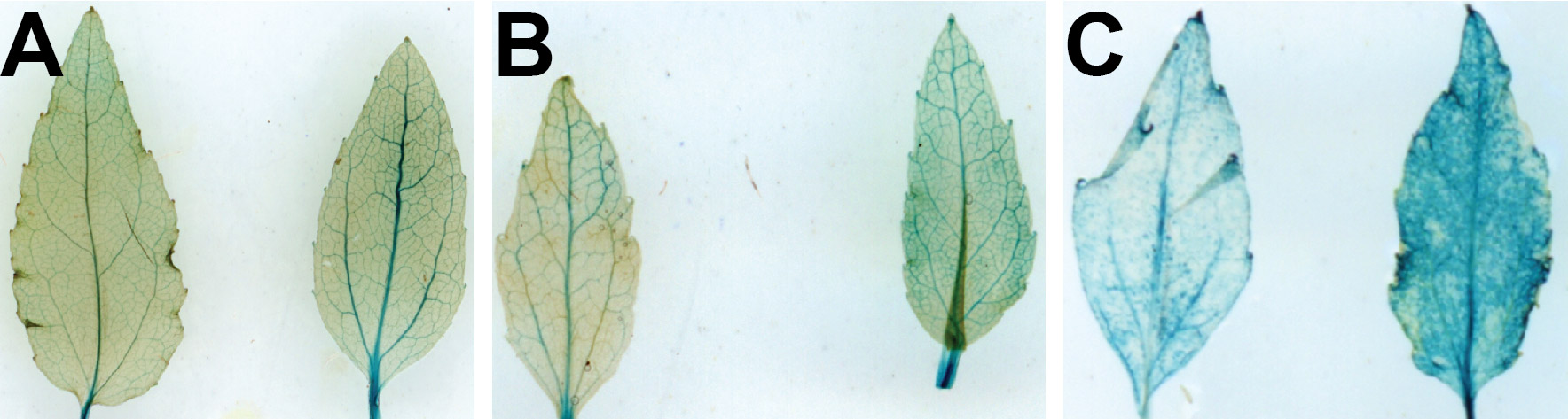

Supplement: Supplementary file 3 [file Image_1.JPEG]
